# Detection of excretory *Entamoeba histolytica *DNA in the urine, and detection of *E. histolytica *DNA and lectin antigen in the liver abscess pus for the diagnosis of amoebic liver abscess

**DOI:** 10.1186/1471-2180-7-41

**Published:** 2007-05-18

**Authors:** Subhash C Parija, Krishna Khairnar

**Affiliations:** 1 Department of Microbiology, Jawaharlal Institute of Postgraduate Medical Education and Research (JIPMER), Puducherry, India

## Abstract

**Background:**

Amoebic liver abscess (ALA) and pyogenic liver abscesses (PLA) appear identical by ultrasound and other imaging techniques. Collection of blood or liver abscess pus for diagnosis of liver abscesses is an invasive procedure, and the procedure requires technical expertise and disposable syringes. Collection of urine is a noninvasive procedure. Therefore, there has been much interest shown towards the use of urine as an alternative clinical specimen for the diagnosis of some parasitic infections. Here, we report for the first time the detection of *E. histolytica *DNA excreted in the urine for diagnosis of the cases of ALA.

**Results:**

*E. histolytica *DNA was detected in liver abscess pus specimen of 80.4% of ALA patients by a nested multiplex polymerase chain reaction (PCR) targeting 16S-like r RNA gene. The nested PCR detected *E. histolytica *DNA in all 37 (100%) liver abscess pus specimens collected prior to metronidazole treatment, but were detected in only 53 of 75 (70.6%) pus specimens collected after therapy with metronidazole. Similarly, the PCR detected *E. histolytica *DNA in 21 of 53 (39.6%) urine specimens of ALA patients. The test detected *E. histolytica *DNA in only 4 of 23 (17.4%) urine specimens collected prior to metronidazole treatment, but were detected in 17 of 30 (56.7%) urine specimens collected after treatment with metronidazole. The enzyme-linked immunosorbent assay (ELISA) for the detection of lectin *E. histolytica *antigen in the liver abscess pus showed a sensitivity of 50% and the indirect haemagglutination (IHA) test for detection of amoebic antibodies in the serum showed a sensitivity of 76.8% for the diagnosis of the ALA.

**Conclusion:**

The present study for the first time shows that the kidney barrier in ALA patients is permeable to *E. histolytica *DNA molecule resulting in excretion of *E. histolytica *DNA in urine which can be detected by PCR. The study also shows that the PCR for detection of *E. histolytica *DNA in urine of patients with ALA can also be used as a prognostic marker to assess the course of the diseases following therapy by metronidazole. The detection of *E. histolytica *DNA in urine specimen of ALA patients provides a new approach for the diagnosis of ALA.

## Background

Infection with *Entamoeba histolytica*, results in 34 million to 50 million symptomatic cases of amoebiasis worldwide each year, causing 40 to 100 thousand deaths annually [1]. Mortality from amoebiasis is mainly due to extra-intestinal pathology, of which amoebic liver abscess (ALA) is the most common. If left untreated, ALA can rupture into neighboring tissue and spread to the brain and other organs via hematological route producing serious morbidity and mortality. It is difficult to differentiate clinically the ALA from pyogenic liver abscess (PLA) as well as from other space occupying lesions of liver such as hydatid cyst and liver hepatoma [2,3].

Imaging techniques like ultrasound, computed tomography, and magnetic resonance although are highly sensitive to detect abscesses in the liver of varied aetiology, however fail to distinguish specifically ALA from that of PLA. Less than one third of patients with ALA have active diarrhea [3]. Hence, stool microscopy and stool antigen detection is not very helpful for diagnosis of ALA. In fact less than only 10% of ALA patients have identifiable *E. histolytica *in stool specimens [4].

Laboratory diagnosis of ALA is usually established by conventional antibody-based serological tests. Nevertheless, the main disadvantage with antibody detection is that serum antibody levels in individuals living in endemic areas, continues to remain positive even for years after infection with *E. histolytica *[5-7]. The demonstration of amoebic antibodies in the serum, therefore, fails to denote the amoebic infection whether it is recent or old. Furthermore, serum amoebic antibodies are not demonstrated in up to 10% of the patients with acute ALA [3].

The demonstration of *E. histolytica *trophozoite in liver abscess pus aspirates by microscopy confirms the diagnosis of ALA, but in best of the laboratories, the amoebic trophozoites can be demonstrated in only 15% of the liver pus [8]. Since the trophozoites of *E. histolytica *are found mainly in the periphery of the abscess diagnosis of ALA by culture of liver pus for *E. histolytica *is also unsatisfactory [9].

Demonstration of amoebic antigen in the liver pus is a recent approach for specific diagnosis of the ALA. A monoclonal antibody-based second generation TechLab enzyme-linked immunosorbent assay (ELISA) kit (Blacksburg, Va.) has been reported to be 78% sensitive for the detection of *E. histolytica *lectin antigen in the liver pus for the diagnosis of ALA in Dhaka, Bangladesh [10]. Studies conducted in various laboratories worldwide including ours have shown that polymerase chain reaction (PCR) is a sensitive and specific method for detecting *Entamoeba *DNA in stool samples and for differentiating the morphologically similar *E. histolytica *from *Entamoeba dispar *and *Entamoeba moshkovskii *[11-19]. However, only few studies using the PCR have been reported for the detection of *Entamoeba *DNA in liver abscess pus for the diagnosis of the ALA [20,21,9,10].

Collection of blood or liver abscess pus is an invasive procedure, and the procedures require technical expertise and disposable syringes [22]. The method if not carried out under stringent conditions is associated with the risk of acquiring needle-borne infections such as hepatitis B virus and human immunodeficiency virus (HIV). Therefore, of late much interest has been shown towards the use of urine as a specimen alternate to the blood for the diagnosis of some parasitic infections including malaria, schistisomiasis, kala-azar, cystic echinococcosis and neurocysticercosis [22]. Urinary antigen for cystic echinococcosis (CE) and neurocysticercosis has been reported for the first time from our laboratory at Jawaharlal Institute of Postgraduate Medical Education and Research (JIPMER), Puducherry, India [23,24]. Our laboratory has developed for the first time a counter-current immunoelectrophoresis (CIEP) and co-agglutination (Co-A) to detect the hydatid antigen excreted in the urine for the diagnosis of CE [23,25], and Co-A to detect cysticercus antigen in the urine for the diagnosis of neurocysticercosis [24].

Detection of DNA in urine by PCR has been employed for the diagnosis of *Toxoplasma gondii*, *Neisseria gonorrhoeae*, *Borrelia burgdorferi*, *Mycobacterium tuberculosis*, *Mycobacterium leprae *and *Chlamydia trachomatis *infections [26-30]. Some studies have also shown that the kidney barrier in rodents and humans is permeable to DNA molecules large enough to be analyzed by standard genetic methodologies [31,32]. To the best of our knowledge till now there is no report available on detection of *Entamoeba *DNA in the urine for the diagnosis of ALA. In the present study, therefore for the first time, we have evaluated a nested multiplex PCR for detection of *Entamoeba *DNA excreted in the urine for the diagnsosis of ALA.

## Results

The quantification of DNA in the liver abscess pus and urine specimen by Spectrophotometric analysis showed the DNA yield to be approximately 85 and 3 μg/ml respectively. The purity of DNA extract from liver abscess pus and urine specimens was found to be satisfactory as the value of ratio of readings at 260 nm and 280 nm (OD_260_/OD_280_) was approximately 1.8–1.85.

The sequencing result of PCR product of *E. histolytica *from liver abscess pus and urine specimen showed 99% identities to the sequence deposited in GenBank, [accession number: X56991]. The result of assessment of competition of other non target DNA present in liver abscess pus (PLA pus negative for *E. histolytica*) and urine (negative control group) specimen with target DNA, showed expected amplification and no nonspecific amplification in nested multiplex PCR.

Estimation of minimum number of *Entamoeba *cells detectable by nested multiplex PCR showed that the detection limit of PCR was found to be approximately 15 *E. histolytica *cells as even 1.5 μl of template DNA from 1000 *E. histolytica *cells/100 μl of Tris- ethylenediamine tetraacetic acid (EDTA) (TE) buffer produced a positive signal.

The IHA test was positive for serum antibodies in the serum of 86 (61.9%) of 139 patients provisionally diagnosed as ALA. The test was positive in a higher number of serum (71 of 102 [69.6%]) samples of patients who had received prior treatment with metronidazole than those who had not received any prior treatment with metronidazole (15 of 37 [40.5%]) and this difference was statistically significant (χ^2 ^= 8.53, P = 0.003). Metronidazole treatment was initiated from a few days to several weeks before collection of the blood samples in these patients. Two (4.6%) out of 43 sera from control cases were positive for antiamoebic antibody by IHA.

The TechLab *E. histolytica *II test was positive for *E. histolytica *Gal/GalNAc lectin antigen in the liver abscess pus of 56 (40.3%) of 139 provisionally diagnosed ALA patients. The TechLab *E. histolytica *II test detected lectin antigen in 30 (81%) of 37 liver abscess pus of patients which were collected prior to treatment with metronidazole. In contrast, the TechLab *E. histolytica *test detected the lectin antigen in only 26 (25.5%) of 102 liver pus (χ^2 ^= 32.61, P < 0.001), collected after initiation of therapy with metronidazole. The probability of *E. histolytica *antigen detection in liver abscess pus by ELISA was found to be 12 times more in patients who had not received prior treatment with metronidazole (Odds Ratio (OR) = 12.53, 95% Confidence Interval (CI) = 4.55 to 35.86) than in the patients who received prior metronidazole therapy. The OR was statistically significant as the 95% CI of OR was more than 1.

Microscopy of the liver pus demonstrated *E. histolytica *trophozoites in 10 of 139 (7.2 %) liver abscess specimens, but only 2 (1.4%) pus specimens were positive by culture for *E. histolytica*. All 10 patients whose liver pus were positive for *E. histolytica *by microscopy and/or culture were also positive for *E. histolytica *Gal/GalNAc lectin antigen in the liver pus by the TechLab *E. histolytica *II test and serum amoebic antibodies by the IHA test.

A total of 102 out of 139 (73.4%) liver abscess pus were negative for aerobic bacteria by Gram's staining and bacterial culture. Twenty seven liver abscess pus specimens were positive for aerobic bacteria by Gram's staining and bacterial culture. These included *Klebsiella pneumoniae *(n = 9), *Proteus *species (n = 5), *Enterobacter *(n = 5), *Escherichia coli *(n = 3) and *Pseudomonas *(n = 5). Ten liver abscess pus specimens showed secondary infection of ALA with aerobic bacteria by Gram's staining and bacterial culture. These included *K. pneumoniae *(n = 3), *Enterobacter *(n = 2), *E. coli *(n = 1), group B *Salmonella *species (n = 1), *Enterococcus *(n = 1) and Coagulase negative *Staphylococci *(n = 2). Such secondary infection of ALA with bacteria has been reported previously in the literature [33,34].

In the present study, a total of 112 out of 139 (80.6%) provisionally diagnosed ALA patients were diagnosed as ALA and remaining 27 patients were diagnosed as PLA, by following the criteria mentioned in this study elsewhere.

**Figure 1:**
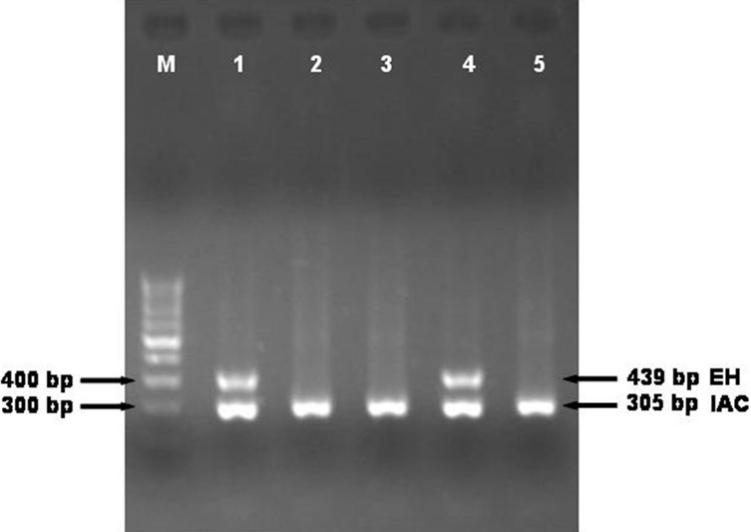
Result of nested multiplex PCR on representative liver abscess pus and urine specimen. The *E. histolytica *(EH) and Internal amplification control (IAC) bands are 439 and 305 bp respectively. Lane-1 and 4 are positive for *E. histolytica *DNA in liver abscess pus and urine specimen respectively; Lane-2, 3 and 5 are negative for *E. histolytica *DNA; Lane-M, 100 bp DNA ladder (Bangalore genei, Bangalore).

The result of nested multiplex PCR performed on the liver abscess pus is depicted in Figure 1. The nested multiplex PCR test was positive for *E. histolytica *DNA in 90 (80.4%) of 112 liver abscess pus specimens (Table 1). The nested multiplex PCR could detect *E. histolytica *DNA in the liver abscess pus of all 37 ALA patients (100%), who were tested prior to treatment with metronidazole. In contrast, prior metronidazole treatment significantly decreased the ability of the PCR to detect *E. histolytica *DNA in the liver abscess pus, with only 53 (70.6%) of 75 liver pus samples positive (Fisher's Exact test, P = 0.0006). The probability of *E. histolytica *DNA detection in liver abscess pus by nested multiplex PCR was 31 times more in patients who had not received prior metronidazole therapy (OR = 31.54, 95% CI = 1.879 to 624.2) than in the patients who received prior metronidazole therapy. The OR was statistically significant as the 95% CI of OR was greater than 1.

The nested multiplex PCR did not detect *E. histolytica *DNA in a total of 49 liver abscess pus specimens, which included 27 PLA and 22 ALA pus specimens. The probability of negative nested multiplex PCR results, in these 49 liver abscess pus specimens due to PCR inhibitors was ruled out by the inclusion of an internal amplification control (IAC) in the nested PCR reaction.

The comparison of results of nested multiplex PCR and TechLab *E. histolytica *II ELISA test on liver abscess pus from ALA patients is summarised in the table 1.

The nested multiplex PCR result on urine specimen is shown in Figure 1. The nested multiplex PCR was performed on urine specimen collected from 68 patients (including 53 ALA and 15 PLA) and 43 controls. The nested multiplex PCR test detected *E. histolytica *DNA in 21 (39.6%) of 53 urine samples collected from patients with ALA (Table 2). The test did not detect *E. histolytica *DNA in urine samples collected from all 15 PLA patients and 43 controls.

The nested multiplex PCR test detected *E. histolytica *DNA in the urine specimens of 4 (17.4%) of 23 ALA patients who were tested prior to treatment with metronidazole and in 17 (56.7%) of 30 ALA patients who were tested after treatment with metronidazole (χ^2 ^= 6.83, P = 0.009). All of the 4 ALA patients, who did not receive prior treatment with metronidazole and whose urine specimens were positive for *E. histolytica *DNA, were available for follow-up study. Urine specimens were collected from these patients every week for 4 weeks after starting of the therapy with metronidazole; and were tested for *E. histolytica *DNA by PCR. It was observed that 2 weeks after treatment with metronidazole, 3 (75%) out of 4 urine specimens became negative for *E. histolytica *DNA. One urine specimen became negative for *E. histolytica *DNA after 4 weeks of treatment with metronidazole.

## Discussion

In this study, we have made an attempt to detect excretory *E. histolytica *DNA in urine by applying nested multiplex PCR and to assess the diagnostic potential of the test for detection of *E. histolytica *DNA in urine for the diagnosis of ALA. Also we have studied the kinetics of the excretion of *E. histolytica *DNA in urine during the course of therapy with metronidazole.

In our study, 76.8% (86 of 112) of ALA patients were positive for antiamoebic antibody in serum by the IHA. This result was similar to that reported from Bangladesh where serum antiamoebic antibodies were found in 78% of ALA patients [10], but differed from that of the study reported from South Africa, where serum antiamoebic antibodies were found in a higher 99% of ALA patients [35].

Only two out of 10 ALA pus samples which were positive for *E. histolytica *trophozoite by microscopy were positive for the amoebae by culture and the rest were negative, this may be due to the inhibition of growth by culture itself. In majority of patients, *K. pneumoniae *was the major bacterial pathogen responsible for PLA and as secondary bacterial infection of ALA. One of 10 ALA pus specimens was positive for Group B *Salmonella *species, this patient had liver abscess with perihepatic collection, with severe sepsis and disseminated intravascular coagulation, finally the patient died. In this study, the anaerobic culture of liver abscess pus aspirate was not done. Therefore, the possible etiology of liver abscess due to anaerobic organisms such as *Bacteroides *remained undetermined.

In the present study, 50% (56 of 112) of liver abscess pus were positive for *E. histolytica *lectin antigen. The sensitivity of the test in our study was observed to be slightly higher than that of the study using the same TechLab *E. histolytica *II kit (40.7 % sensitiivty) on liver pus reported from Bangladesh [10]. However, results of other studies using polyclonal antibody based ELISA showed a very high sensitivity for the detection of amoebic antigen in the liver pus. Amoebic antigen was detected in liver abscess pus in 97.6% (41/42) of ALA cases by ELISA as reported from China [21] and in 92% and 96% of liver pus by using immunoelectrophoresis and ELISA respectively, from India [36].

In developing countries like India where amoebiasis is endemic, antiamoebic drugs and antibiotics are used indiscriminately, making it difficult to obtain an accurate treatment history. Most of the patients in the present study had already been treated with metronidazole at the time of collection of clinical specimens. The serum amoebic antibodies were detected in higher percentage (94.7%) of ALA patients treated earlier with metronidazole, but were detected in only 40.5% of patients who did not receive any prior treatment with metronidazole. This might be due to the late antibody response during the course of the disease.

Unlike serum amoebic antibody detection, *E. histolytica *lectin antigen was detected in liver pus by TechLab ELISA in a higher percentage (81%) of ALA patients, who were tested prior to treatment with metronidazole, but was detected in only 34.6% of ALA patients, who were tested after the initiation of therapy with metronidazole. This might be due to the rapid clearing of amoebic antigen from the liver pus due to killing of *E. histolytica *trophozoites on treatment with metronidazole.

The PCR for the detection of *E. histolytica *DNA in liver abscess pus had a much higher sensitivity (100%) when tested prior to treatment with metronidazole, but had a lower sensitivity (70.6%) when tested after the initiation of treatment with metronidazole. This might be attributed to the clearing of *E. histolytica *DNA from the liver abscess due to the death and lysis of *E. histolytica *trophozoites on treatment with metronidazole. The percentage of agreement between *E. histolytica *DNA detection and ELISA for *E. histolytica *antigen detection in liver abscess pus was found to be 67.9% in the present study (Table 1). The Kappa statistic was found to be 0.36 which indicates a fair agreement between the two tests.

PCR for detection of *E. histolytica *DNA and ELISA for detection of *E. histolytica *lectin antigen in liver abscess pus were evaluated for the diagnosis of ALA (McNemar's χ^2 ^= 30.25, p < 0.0001). The sensitivity of PCR was 80.4%. This was found to be significantly higher than that of ELISA (50 %) using McNemar's χ^2 ^test (p < 0.0001). All 27 liver abscess pus aspirates diagnosed as PLA were negative for *E. histolytica *DNA by PCR and for *E. histolytica *lectin antigen by TechLab ELISA, which represents a specificity of 100%. ELISA for detection of liver abscess pus *E. histolytica *lectin antigen demonstrated a 100% positive predictive value and a 32.5% negative predictive value. PCR for the detection of liver abscess pus *E. histolytica *DNA demonstrated a 100% positive predictive value and a 55.1% negative predictive value.

In the present study *E. histolytica *DNA was detected in the urine specimen of 4 (17.4%) of 23 ALA patients, who were tested prior to treatment with metronidazole and in 17 (56.7%) of 30 ALA patients, who were tested after treatment with metronidazole by PCR. The probability of *E. histolytica *DNA detection in urine by PCR was 6 times more in ALA patients who had received prior metronidazole therapy (OR = 6.2, 95% CI = 1.47 to 28.37) than in the ALA patients who did not receive prior metronidazole therapy. The OR was statistically significant as the 95% CI of OR was greater than 1. This might be due to release of increased *E. histolytica *DNA from the dying *E. histolytica *trophozoites when metronidazole therapy was initiated, leading to excretion of *E. histolytica *DNA in the urine. One study has demonstrated that the DNA from dying cells can cross the kidney barrier in rodents and humans and can get excreted with urine, which can be used for genetic analysis [31].

PCR for detection of *E. histolytica *DNA in liver abscess pus and urine specimen were evaluated for the diagnosis of ALA (McNemar's χ^2 ^= 26.28, p < 0.0001). The sensitivity of PCR for urine was 39.6%. This was found to be significantly lower than that of PCR for liver abscess pus (80.4%) using McNemar's χ^2 ^test (p < 0.0001). All urine specimens from 15 PLA patients and 43 control group individuals were negative for *E. histolytica *DNA by PCR. This represents a specificity of 100% (Table 2). PCR for the detection of urinary *E. histolytica *DNA demonstrated a 100% positive predictive value and a 31.9% negative predictive value.

As per the PCR kinetics the likelihood of amplifying smaller PCR product is more than amplifying larger PCR product. We feel that if the PCR product smaller than 400 bp would have been amplified, the PCR might show higher sensitivity.

*E. histolytica *DNA in urine did not persist longer in ALA patients after treatment with metronidazole as observed in the present study. Three of 4 (75%) urine specimens positive for *E. histolytica *DNA became negative for *E. histolytica *DNA within 2 weeks of treatment with metronidazole. This might be attributed to the reduced excretion of *E. histolytica *DNA in the urine as a result of reduction of *E. histolytica *infection load following treatment with metronidazole. The effect of metronidazole in killing of *E. histolytica *and clearing of antigenemia in hamsters suffering from hepatic amoebiasis has been well documented in a study reported by Thammapalerd et al. [37] Results of the present study therefore indicate that the PCR can be used to monitor excretion of *E. histolytica *DNA in urine as a prognostic marker during therapy of ALA with specific antiamoebic drugs.

In the present study, none of the liver abscess pus and urine PCR results were positive for either *E. dispar *or *E. moshkovskii *specific PCR products, which confirm the non invasive nature of these species (Table 2).

The detection of *E. histolytica *DNA and *E. histolytica *specific lectin antigen in the serum specimen of ALA patients was not carried out in this study. A controlled prospective study to evaluate the detection of *E. histolytica *DNA and *E. histolytica *specific lectin antigen in the serum specimen of ALA patients has been intended to be carried out in future in our laboratory.

## Conclusion

The present study for the first time shows that the kidney barrier in ALA patients is permeable to *E. histolytica *DNA molecule resulting in excretion of *E. histolytica *DNA in urine which can be detected by PCR. The study also shows that the PCR for detection of *E. histolytica *DNA in urine of patients with ALA can also be used as a prognostic marker to assess the course of the diseases following therapy by metronidazole. The detection of *E. histolytica *DNA in urine specimen of ALA patients provides a new approach for the diagnosis of ALA.

## Methods

### Sample details

The subjects in the present study included 139 patients provisionally diagnosed as ALA, who were admitted to JIPMER hospital, Puducherry, as well as 43 controls during a period from September 2004 to March 2006. The provisional diagnosis of ALA was made by the physicians on the basis of patient's history and clinical features, unfortunately these features are often nonspecific to confirm the diagnosis of ALA. Of the 139 provisionally diagnosed ALA patients, 102 had received prior treatment and 37 did not receive prior treatment with metronidazole. The patients and controls were residing in neighboring area of Puducherry. Informed consent was obtained from the patients. This study has been performed with the approval of Institute Research Council of JIPMER, Puducherry.

The control group included 43 individuals who had no history of recent dysentery or diarrhea and whose stool samples were negative for *E. histolytica *infection by microscopy and culture. Thirty five of the controls were healthy asymptomatic volunteers, and the other 8 patients included, hydatid cyst in liver (n = 2), liver hepatoma (n = 1), liver cirrhosis (n = 3), and viral hepatitis (n = 2).

The diagnosis of ALA was established on the basis of radiological, symptomatological and laboratory criteria as follows [3,10]: (i) a space-occupying lesion in the liver diagnosed by ultra sonography and suggestive of abscess, (ii) clinical symptoms (fever, pain in the right hypochondrium (often referred to the epigastrium), lower chest, back, or tip of the right shoulder), (iii) enlarged and/or tender liver, usually without jaundice, (iv) raised right dome of the diaphragm on chest radiograph, (v) improvement after treatment with antiamoebic drugs (e.g., metronidazole) (vi) positive IHA of serum antibody showing a titer (≥ 1:128) against *E. histolytica*, (vii) liver aspirate appeared like anchovy sauce but was bacteriologically sterile.

### Sample collection

Liver abscess pus: The aspiration of liver abscess pus was indicated only under the following conditions [3]. (i) to rule out a pyogenic abscess; (ii) the failure to respond clinically in 3 to 5 days; (iii) the threat of imminent rupture; and (iv) the prevention of rupture of left-lobe abscess into the pericardium. The liver abscess pus aspirates were performed, only for clinical purposes as judged by the clinicians for the patient care and not for the purpose of this study. Liver abscess pus was obtained under ultrasound guidance from all 139 provisionally diagnosed ALA patients and was stored at -20°C in a sterile container until used.

Urine: Urine specimen could be collected from 68 out of 139 provisionally diagnosed ALA patients and all 43 control group individuals included in the study. 10 ml of urine specimen was collected in a sterile container using aseptic techniques; urine sample was stored at -20°C until use.

Blood: Blood specimen was collected from all 139 provisionally diagnosed ALA patients and 43 control group individuals included in the study. Venous blood (5 ml) was collected in a sterile container; sera were separated and stored at -20°C until used.

### Microscopy for *Entamoeba*

Liver abscess pus: The specimens were examined immediately after the aspiration of abscess. The liver abscess pus was first centrifuged at 2,500 g for 5 mins and a loopful (usually inoculating needle loop) of sediment was mixed with a drop of warm saline on a microscope slide. Microscopic examination of an amoebic abscess aspirate from liver may reveal haematophagous trophozoites.

### Culture for *Entamoeba*

Liver abscess pus: Liver abscess pus specimens were cultured for *Entamoeba *species in Locke-egg (LE) medium (NIH modification of Boeck and Drbohlav's medium) as previously described [38]. The liver abscess pus was first centrifuged at 2,500 g for 5 mins and a loopful of sediment was inoculated into the LE medium. It is to be noted that in case of culturing *Entamoeba *from liver abscess aspirates, since the abscess is sterile, bacterial flora (ATCC *E. coli*) was added before inoculation of amoebae into xenic culture.

### Gram stain and bacterial culture for liver abscess pus aspirates

Direct smear Gram staining and bacterial culture was done for all liver abscess pus aspirates. The liver abscess pus specimens were inoculated on to sheep blood agar, MacConkey agar and chocolate agar plates. The MacConkey agar plates were incubated at 37°C for 18–24 hours whereas the blood agar and chocolate agar plates were incubated in a candle jar at 37°C for 48 hours. Based on the colony morphology and result of culture smears, necessary biochemical tests were done to identify bacteria to the species level.

### TechLab *E. histolytica *II ELISA test

The TechLab *E. histolytica *II test was performed on liver abscess pus specimens to detect *E. histolytica *as per the method described earlier [10].

### Antiamoebic antibody detection by rapid-IHA test

The Rapid-IHA was performed on serum specimen as per the method described earlier [39]. Briefly, double aldehyde stabilized chick red blood cell (RBC) sensitized with the amoebic antigen was used in the test. The haemagglutination test was performed on test serum samples including known positive and negative control sera in each batch. The chick RBC settled quickly and their haemagglutination pattern could be determined within 30 to 45 min of incubation at room temperature with test sera [39]. A titer of ≥ 1:128 was considered positive for ALA [40].

### Extraction of *Entamoeba *genomic DNA

Liver abscess pus: Briefly, 1 ml of liver abscess pus was taken in 1.5 ml centrifuge tube and centrifuged at 12, 000 g for 8 minutes. The supernatant was discarded and 50 μl of pellet was dispersed in 250 μl of lysis buffer (0.25% sodium dodecyl sulfate (SDS) in 0.1 M EDTA, pH 8.0), followed by addition of 100 μg/ml of proteinase K. The lysate was incubated at 55 °C for 2 hours. Then 75 μl of 3.5 M sodium chloride (NaCl) followed by 42 μl of 10% cetyltrimethylammonium bromide (CTAB)/0.7 M NaCl (heated to 55 °C) was added. After the components were mixed, the sample was incubated at 65°C for 30 min. This was followed by extractions with equal volumes of chloroform and then phenol-chloroform-isoamyl alcohol, and the DNA was precipitated with ice cold ethanol. The dried DNA pellet was dissolved in 50 μl of sterile distilled water.

Urine: Briefly, 10 ml of the urine sample was centrifuged at 12,000 g for 15 min at 4 °C. The supernatant was discarded and the pellet was suspended in 500 μl of sterile distilled water. The suspension was boiled for 10 minutes followed by sudden cooling. Next, 5 μl of proteinase-K (10 mg/ml) and 60 μl of 10% SDS were added to the suspension and incubated at 65 °C for three hours. Then 80 μl of 5 M NaCl and 20 μl of 10% CTAB were added to the mixture and incubated at 65 °C for 45 min. This was followed by extractions with equal volumes of chloroform and then phenol-chloroform-isoamyl alcohol. The DNA was precipitated with ice cold ethanol. The dried DNA pellet was dissolved in 50 μl of sterile distilled water.

The protocol for extraction of DNA from liver abscess pus and urine specimen has been modified in our laboratory from CTAB DNA extraction protocol originally described for DNA extraction from amoebic culture [41]. The extracted DNA from liver abscess pus and urine sample was passed through DNA clean-up spin columns (Bangalore Genei KT-62, Bangalore). The DNA was stored at -20 °C until used.

### Quantification of DNA in liver abscess pus and urine specimen

DNA quantification in spin column purified DNA extract from liver abscess pus and urine specimen was determined by UV absorbance using a Cintra 5 double beam Spectrophotometer. DNA yields were calculated on the basis of UV absorbance × dilution. The purity of the nucleic acid in the samples was estimated by the ratio of readings at 260 nm and 280 nm (OD_260_/OD_280_).

### Primers design

The genus specific primers were designed using nucleotide sequences of 16S-like rRNA gene of *E. dispar*, *E. histolytica *and *E. moshkovskii *Laredo deposited in GenBank [accession number : Z49256], [accession number : X56991] and [accession number : AF149906] respectively.

The comparison of all the three 16S-like rRNA gene sequences of *E. dispar*, *E. histolytica *and *E. moshkovskii *Laredo revealed significant differences enough to design species specific primers. In this study, we have used an IAC targeting human 18S ribosomal RNA gene to rule out false-negative results in clinical specimens. All the primers were designed using Prime3 online software [42]. The primers used in PCR are shown in Table 3.

### Standard strains

Three standard strains used in this study were *E. histolytica *HM-1: IMSS, *E. dispar *SAW760, and *E. moshkovskii *Laredo these were used as positive control in the present study. The lyophilized DNA of these strains was generously gifted by Dr. C. Graham Clark from London School of Hygiene & Tropical Medicine, London, UK.

### Nested multiplex PCR protocol

Liver abscess pus PCR: For a reaction volume of 25 μl, comprising 2.5 μl of 10X PCR buffer (Biogene), 2.0 μl of 25 mM MgCl_2 _(Bangalore genei), 0.75 μl of deoxynucleoside triphosphate mix (10 mM each dNTP, Biogene), 0.3 μl (5 IU/μl) of *Taq *polymerase (Biogene), 10 picomoles of target DNA primers (IDT) and 5 picomoles of IAC primers (IDT) were added in genus and species specific PCR. The template DNA volume was 2 μl for both genus and species specific PCR. The PCR tubes were finally placed in an Eppendorf Thermal cycler [Master cycler gradient].

Urine PCR: The PCR mix composition was the same as described earlier for liver abscess pus PCR, except that 1.0 μl of 25 mM MgCl_2 _and 2.5 μl of template DNA was added.

The conditions for genus specific PCR were as follows; the PCR mix was subjected to an initial denaturation at 96 °C for 2 minutes, followed by 30 cycles – each consisting of 92 °C for 60 seconds (Denaturation), 56 °C for 60 seconds (Annealing), and 72 °C for 90 seconds (Extension). Finally one cycle of extension at 72 °C for seven minutes was performed. In the species specific nested multiplex PCR (which had multiple primer sets in the same tube), only the annealing temperature was changed to 48 °C, leaving the other parameters of the amplification cycles unchanged.

3.5 μl of the amplification product was separated by electrophoresis through 1.8% agarose gel (Agarose Low EEO, Bangalore genie products, Bangalore, India) containing ethidium bromide in 0.5 × Tris-borate-EDTA at 120 V for 45 min and was visualized under UV light for bands of DNA of appropriate sizes (Figure 1). A negative control reaction was included with each batch of samples analyzed by PCR.

### Primer validation

The primer sequences designed for *E. moshkovskii*, *E. histolytica*, *E. dispar *and Human IAC were subjected to Basic Local Alignment Search Tool (BLAST) in the genome database of all organisms [43] and were found to be specific for the study. The amplified PCR products of *E. histolytica *species in liver abscess pus and urine samples was confirmed by getting both the strands of DNA sequenced on ABI3730XL sequencer (Macrogen, Seoul, South Korea). The sequencing was done using species specific primers i.e. EH-1/EH-2 for *E. histolytica*. All sequences were analyzed for homology by using the nucleotide-nucleotide BLAST search feature [43].

The identity between the sequencing results of PCR product of *E. histolytica *from liver abscess pus and urine with the sequence deposited in GenBank [accession number: X56991] were analyzed by using the "Align two sequences (bl2seq)" feature [43].

### Assessment of competition of non target DNA

During the standardization to assess the competition of other non target DNA present in liver abscess pus and urine specimen with target DNA, the nested multiplex PCR was checked with reference DNA (DNA from standard culture of *E. histolytica*, *E. dispar *&*E. moshkovskii*) spiked with DNA from liver abscess pus (PLA pus negative for *E. histolytica*) and urine (negative control group) followed by nested multiplex PCR amplification.

### Estimation of minimum number of *Entamoeba *cells detectable by nested multiplex PCR

This was performed for *E. histolytica *with Locke-egg (LE) medium (NIH modification of Boeck and Drbohlav's medium) liver abscess pus cultures; the amoebae were counted using a standard haemocytometer. A cell pellet containing 10^6 ^cells was preferred for determining the detection limit of nested multiplex PCR for *E. histolytica*. The cell pellet containing 10^6 ^cells of *E. histolytica *was diluted 10 folds in phosphate buffer saline (PBS) to obtain different concentration of cells, such as 10^5^, 10^4^, 10^3^, 10^2 ^and 10 cells/ml. The different dilutions of cells ranging from 10^6 ^to 10 cells/ml were centrifuged and the remaining pellet of each dilution was added to 0.05 μl of liver abscess pus (PLA pus negative for *E. histolytica*) followed by DNA extraction and PCR as per the aforementioned protocol.

### Statistical data analysis

Sensitivity was calculated as follows: number of patients with positive test results/total number of patients × 100. Specificity was calculated as follows: number of controls with negative test results/total number of controls × 100. The positive predictive value was calculated as follows: number of true positives/(number of true positives + number of false positives) × 100. The negative predictive value was calculated as follows: number of true negatives/(number of true negatives + number of false negatives) × 100. The agreement between the tests was calculated using the Kappa statistics. To determine the statistical significance of differences between the proportions, Chi-square (χ^2^) test and Fisher's exact test were used. The χ^2 ^test and odds ratio were found using "Epi Info Version 6". To calculate the significance of the difference in sensitivities, McNemar's Chi-square test was applied. The McNemar's test was performed using "Graph Pad Software".

## Authors' contributions

SCP supervised and coordinated the study, and helped to draft the manuscript. KK carried out the experimental works, and drafted the manuscript.

## Figures and Tables

**Table 1:** Comparison of result of PCR and antigen detection in liver abscess pus specimen of ALA patients.

PCR results	Antigen detection result (no. of specimens positive)		
		
	*E. histolytica*	Antigen negative	Total no. of specimens
*E. histolytica*	55^*a*^	35	90
Negative	1	21	22
Total	56	56	112

^*a *^*E. histolytica *was detected by microscopy and/or culture in 10 of these 55 ELISA and PCR positive liver abscess pus specimens.

**Table 2:** Detection of serum antiamoebic antibody, liver pus *E. histolytica *antigen, liver pus *Entamoeba *DNA and urine *Entamoeba *DNA in ALA patients.

		No. (%) of positive results by:							
		
		IHA for :	ELISA for:	PCR for:					
					
Diagnosis	No of patients								
		Antiamoebic antibody in sera	Amoebic lectin antigen in liver pus	*Entamoeba *DNA in liver pus			*Entamoeba *DNA in urine		
					
				*E. histolytica*	*E. dispar*	*E. moshkovskii*	*E. histolytica*	*E. dispar*	*E. moshkovskii*
				(439 bp)	(174 bp)	(553 bp)	(439 bp)	(174 bp)	(553 bp)
ALA^a^	53	38 (71.7)	29 (54.7)	51 (96.2)	0	0	21(39.6)	0	0
PLA^b^	15	0	0	0	0	0	0	0	0

^a^amoebic liver abscess; ^b^pyogenic liver abscess

**Table 3:** Primer sequence used in PCR.

Genus specific primers (First PCR)	
*Entamoeba *genus	E-1 5' TAAGATGCACGAGAGCGAAA 3' (forward primer) E-2 5' GTACAAAGGGCAGGGACGTA 3' (reverse primer)

Species specific primers (Second nested multiplex PCR)	

*E. histolytica *species	EH-1 5' AAGCATTGTTTCTAGATCTGAG 3' (forward primer) EH-2 5' AAGAGGTCTAACCGAAATTAG 3' (reverse primer)
*E. moshkovskii *species	Mos-1 5' GAAACCAAGAGTTTCACAAC 3' (forward primer) Mos-2 5' CAATATAAGGCTTGGATGAT 3' (reverse primer)
*E. dispar *species	ED-1 5' TCTAATTTCGATTAGAACTCT 3' (forward primer) ED-2 5' TCCCTACCTATTAGACATAGC 3' (reverse primer)

Internal amplification control (IAC) primer for PCR	

Human 18S ribosomal RNA gene	IAC-1 5' GGCTTTGGTGACTCTAGATA 3' (forward primer) IAC-2 5' CGTTAAAGGATTTAAAGTGG 3' (reverse primer)

## References

[B1] World Health Organization (1997). Amoebiasis. WHO Weekly Epidemiol Rec.

[B2] Smoger SH, Mitchell CK, McClave SA (1998). Pyogenic liver abscesses: a comparison of older and younger patients. Age Ageing.

[B3] Kasper DL, Braunwald E, Fauci AS, Hauser SL, Longo DL, Jameson JL (2005). Harrison's principles of internal medicine.

[B4] Katzenstein D, Rickerson V, Braude (1982). New concepts of amebic liver abscess derived from hepatic imaging, serodiagnosis and hepatic enzymes in 67 consecutive cases in San Diego. Medicine (Baltimore).

[B5] Gandhi BM, Irshad M, Chawla TC, Tandon BN (1987). Enzyme linked protein A: an ELISA for detection of amoebic antibody. Trans R Soc Trop Med Hyg.

[B6] Jackson TF, Gathiram V, Simjee AE (1984). Serological differentiation between past and present infections in hepatic amoebiasis. Trans R Soc Trop Med Hyg.

[B7] Yang J, Kennedy MT (1979). Evaluation of enzyme-linked immunosorbent assay for the serodiagnosis of amoebiasis. J Clin Microbiol.

[B8] Parija SC (1993). Recent trends in the diagnosis of amoebiasis. J Assoc Physicians India.

[B9] Zaman S, Khoo J, Ng SW, Ahmed R, Khan MA, Hussain R, Zaman V (2000). Direct amplification of *Entamoeba histolytica *DNA from amoebic liver abscess pus using polymerase chain reaction. Parasitol Res.

[B10] Haque R, Mollah NU, Ali IK, Alam K, Eubanks A, Lyerly D, Petri WA (2000). Diagnosis of amebic liver abscess and intestinal infection with the TechLab *Entamoeba histolytica *II antigen detection and antibody tests. J Clin Microbiol.

[B11] Hamzah Z, Petmitr S, Mungthin M, Leelayoova S, Chavalitshewinkoon-Petmitr P (2006). Differential detection of *Entamoeba histolytica, Entamoeba dispar*, and *Entamoeba moshkovskii *by a single-round PCR assay. J Clin Microbiol.

[B12] Fotedar R, Stark D, Beebe N, Marriott D, Ellis J, Harkness J (2007). PCR detection of *Entamoeba histolytica, Entamoeba dispar*, and *Entamoeba moshkovskii *in stool samples from Sydney, Australia. J Clin Microbiol.

[B13] Lebbad M, Svard SG (2005). PCR differentiation of *Entamoeba histolytica *and *Entamoeba dispar *from patients with amoeba infection initially diagnosed by microscopy. Scand J Infect Dis.

[B14] Roy S, Kabir M, Mondal D, Ali IK, Petri WA, Haque R (2005). Real-time-PCR assay for diagnosis of *Entamoeba histolytica *infection. J Clin Microbiol.

[B15] Freitas MA, Vianna EN, Martins AS, Silva EF, Pesquero JL, Gomes MA (2004). A single step duplex PCR to distinguish *Entamoeba histolytica *from *Entamoeba dispar*. Parasitology.

[B16] Verweij JJ, Blange RA, Templeton K, Schinkel J, Brienen EA, van Rooyen MA, van Lieshout L, Polderman AM (2004). Simultaneous detection of *Entamoeba histolytica, Giardia lamblia*, and *Cryptosporidium parvum *in fecal samples by using multiplex real-time PCR. J Clin Microbiol.

[B17] Nunez YO, Fernandez MA, Torres-Nunez D, Silva JA, Montano I, Maestre JL, Fonte L (2001). Multiplex polymerase chain reaction amplification and differentiation of Entamoeba histolytica and *Entamoeba dispar *DNA from stool samples. Am J Trop Med Hyg.

[B18] Ali IK, Hossain MB, Roy S, Ayeh-Kumi PF, Petri WA, Haque R, Calrk CG (2003). *Entamoeba moshkovskii *infections in children, Bangladesh. Emerg Infect Dis.

[B19] Parija SC, Khairnar K (2005). *Entamoeba moshkovskii *and *Entamoeba dispar *associated Infections in Pondicherry, India. J Health Popul Nutr.

[B20] Khan U, Mirdha BR, Samantaray JC, Sharma MP (2006). Detection of *Entamoeba histolytica *using polymerase chain reaction in pus samples from amebic liver abscess. Indian J Gastroenterol.

[B21] Zengzhu G, Bracha R, Nuchamowitz Y, Cheng-I W, Mirelman D (1999). Analysis by enzyme-linked immunosorbent assay and PCR of human liver abscess aspirates from patients in China for *Entamoeba histolytica*. J Clin Microbiol.

[B22] Parija SC (1998). Urinary antigen detection for diagnosis of parasitic infections. Parasitol Today.

[B23] Parija SC, Ravinder PT, Subba Rao KSVK (1997). Detection of hydatid antigen in urine by counter-current immunoelectrophoresis. J Clin Microbiol.

[B24] Parija M, Biswas R, Harish BN, Parija SC (2004). Detection of specific cysticercus antigen in the urine for diagnosis of neurocysticercosis. Acta Trop.

[B25] Ravinder PT, Parija SC, Rao KS (2000). Urinary hydatid antigen detection by coagglutination, a cost-effective and rapid test for diagnosis of cystic echinococcosis in a rural or field setting. J Clin Microbiol.

[B26] Crotchfelt KA, Welsh LE, DeBonville D, Rosenstraus M, Quinn TC (1997). Detection of *Neisseria gonorrhoeae *and *Chlamydia trachomatis *in genitourinary specimens from men and women by a coamplification PCR assay. J Clin Microbiol.

[B27] Pleyer U, Priem S, Bergmann L, Burmester G, Hartmann C, Krause A (2001). Detection of *Borrelia burgdorferi *DNA in urine of patients with ocular Lyme borreliosis. Br J Ophthalmol.

[B28] Fuentes I, Rodriguez M, Domingo CJ, del Castillo F, Juncosa T, Alvar J (1996). Urine sample used for congenital toxoplasmosis diagnosis by PCR. J Clin Microbiol.

[B29] Aceti A, Zanetti S, Mura MS, Sechi LA, Turrini F, Saba F, Babudieri S, Mannu F, Fadda G (1999). Identification of HIV patients with active pulmonary tuberculosis using urine based polymerase chain reaction assay. Thorax.

[B30] Parkash O, Singh HB, Rai S, Pandey A, Katoch VM, Girdhar BK (2004). Detection of *Mycobacterium leprae *DNA for 36kDa protein in urine from leprosy patients: a preliminary report. Rev Inst Med Trop Sao Paulo.

[B31] Botezatu I, Serdyuk O, Potapova G, Shelepov V, Alechina R, Molyaka Y, Ananev V, Bazin I, Garin A, Narimanov M, Knysh V, Melkonyan H, Umansky S, Lichtenstein A (2000). Genetic analysis of DNA excreted in urine: a new approach for detecting specific genomic DNA sequences from cells dying in an organism. Clin Chem.

[B32] Su YH, Wang M, Brenner DE, Ng A, Melkonyan H, Umansky S, Syngal S, Block TM (2004). Human urine contains small, 150 to 250 nucleotide-sized, soluble DNA derived from the circulation and may be useful in the detection of colorectal cancer. J Mol Diagn.

[B33] Gathiram V, Simjee AE, Bhamjee A, Jackson TF, Pillai LV (1984). Concomitant and secondary bacterial infection of the pus in hepatic amebiasis. S Afr Med J.

[B34] Sharma MP, Daarathy S, Sushma S, Verma N (1997). Variants of amoebic liver abscess. Arch Med Res.

[B35] Gathiram V, Jackson TF (1987). A longitudinal study of asymptomatic carriers of pathogenic zymodemes of *Entamoeba histolytica*. S Afr Med J.

[B36] Bhave GG, Wagle NM, Joshi UM (1985). Detection of amoebic antigen by enzyme linked immunosorbent assay (ELISA). J Postgrad Med.

[B37] Thammapalerd N, Sherchand JB, Kotimanusvani D, Chintana T, Tharavanij S (1996). Monoclonal antibody-based ELISA for the detection of circulating *Entamoeba histolytica *antigen in hepatic amebiasis in hamsters. Southeast Asian J Trop Med Public Health.

[B38] Parija SC, Rao RS (1995). Stool culture as a diagnostic aid in the detection of *Entamoeba histolytica *in the faecal specimens. Indian J Pathol Microbiol.

[B39] Parija SC, Kasinathan S, Rao RS (1989). Rapid indirect haemagglutination (Rapid-IHA) using sensitised chick cells for serodiagnosis of amoebiasis at primary health centre level. J Trop Med Hyg.

[B40] Parija SC, Kasinathan S, Rao RS (1988). Comparative evaluation of polyxenic and axenic amoebic antigens in indirect haemagglutination (IHA) for serodiagnosis of amoebiasis. Indian J Med Microbiol.

[B41] Clark CG, Diamond LS (1991). The Laredo strain and other *Entamoeba histolytica*-like amoebae are *Entamoeba moshkovskii*. Mol Biochem Parasitol.

[B42] Primer3 Input. http://frodo.wi.mit.edu/.

[B43] BLAST: Basic Local Alignment and Search Tool. http://www.ncbi.nlm.nih.gov/blast/.

